# Improvements in Disease Activity Partially Mediate the Effect of Tofacitinib Treatment on Generic and Disease-Specific Health-Related Quality of Life in Patients with Ulcerative Colitis: Data from the OCTAVE Program

**DOI:** 10.1159/000528788

**Published:** 2023-01-05

**Authors:** Marla C. Dubinsky, Alessandro Armuzzi, Krisztina B. Gecse, Thomas Ullman, Andrew G. Bushmakin, Marco DiBonaventura, Joseph C. Cappelleri, Susan B. Connelly, John C. Woolcott, Leonardo Salese

**Affiliations:** ^a^Susan and Leonard Feinstein IBD Center, Icahn School of Medicine at Mount Sinai, New York, New York, USA; ^b^IBD Unit, IRCCS Humanitas Research Hospital, Rozzano, Milan, Italy; ^c^Department of Gastroenterology and Hepatology, Amsterdam University Medical Center, Academic Medical Center, Amsterdam, The Netherlands; ^d^Division of Gastroenterology, Montefiore Medical Center, Albert Einstein College of Medicine, Bronx, New York, USA; ^e^Pfizer Inc, Groton, Connecticut, USA; ^f^Pfizer Inc, New York, New York, USA; ^g^Pfizer Inc, Collegeville, Pennsylvania, USA

**Keywords:** Gastroenterology, Health-related quality of life, Inflammatory bowel disease, Patient-reported outcomes, Statistical modeling, Ulcerative colitis

## Abstract

**Background:**

Patients with ulcerative colitis (UC) often report impaired health-related quality of life (HRQoL). Tofacitinib is an oral small molecule Janus kinase inhibitor for the treatment of UC. In addition to previous demonstrations of improved clinical measures (e.g., Mayo score), tofacitinib has been shown to improve HRQoL in patients with UC. This analysis explored the interrelationships among tofacitinib treatment, HRQoL, and disease activity (measured using Mayo subscores) using mediation modeling.

**Methods:**

Data were collected from two 8-week induction studies (OCTAVE Induction 1 and 2) in patients with moderate to severe UC treated with tofacitinib or placebo. Two mediation models were specified. First, Mayo subscores were mediators between the binary treatment variable (tofacitinib vs. placebo) and the eight Short Form-36 Health Survey (SF-36) domain scores as outcomes. Second, the four Inflammatory Bowel Disease Questionnaire (IBDQ) domain scores served as outcomes. Both models used data collected at week 8.

**Results:**

Overall, 1,073 and 1,079 patients were included in the SF-36- and IBDQ-based models, respectively. For all SF-36 domains, improvements in Mayo subscores were estimated to explain 65.6% (bodily pain) to 92.9% (mental health) of the total treatment effect on SF-36 domain scores (all *p* < 0.05). For all IBDQ domains, improvements in Mayo subscores explained 71.6% (systemic symptoms) to 84.7% (emotional function) of the total treatment effect (all *p* < 0.05).

**Conclusion:**

Mayo scores and Mayo subscores are significant but incomplete contributors to tofacitinib's effect on HRQoL in patients with moderate to severe UC. ClinicalTrials.gov: NCT01465763; NCT01458951.

## Introduction

Ulcerative colitis (UC) is a chronic inflammatory disease associated with periods of relapse and remission that exerts a significant disease burden, affecting many patients worldwide [1, 2, 3]. Patients with UC often report impaired health-related quality of life (HRQoL) that can result in decreased productivity at work or school or impose limitations on social activities and relationships [4]. Along with clinical response, restoration or normalization of HRQoL is an important goal of UC treatment [5, 6, 7].

Treatment of UC traditionally follows a step-up approach and is dependent on the level of disease activity and extent of colonic involvement [8, 9]. Conventional agents include 5-aminosalicylic acid, corticosteroids, and immunomodulators (e.g., thiopurines). Patients who do not respond to conventional treatment may be treated with biologic therapies, such as tumor necrosis factor inhibitors, anti-integrins, and anti-interleukin-23 treatments [10]. Novel therapies have emerged for the treatment of UC in patients who are intolerant to tumor necrosis factor inhibitors or in whom treatment has failed. These include sphingosine-1-phosphate receptor modulators [11] and Janus kinase (JAK) inhibitors − small molecules that impact the inflammatory process by working intracellularly to interfere with cytokine signaling pathways [12, 13, 14, 15].

Tofacitinib is an oral small-molecule JAK inhibitor for the treatment of UC. Tofacitinib treatment was shown to clinically and statistically significantly improve Mayo score-based outcomes, as well as Short Form-36 Health Survey (SF-36) and Inflammatory Bowel Disease Questionnaire (IBDQ) domain scores, versus placebo, in two 8-week induction studies (OCTAVE Induction 1 and 2, NCT01465763 and NCT01458951) and a 52-week maintenance study (OCTAVE Sustain, NCT01458574) in patients with moderate to severe UC [14, 16]. The Mayo score, a combination of physician- and patient-reported outcomes that measure UC disease activity, is widely used in clinical trials and comprises four subscores: stool frequency, rectal bleeding, endoscopic appearance, and Physician Global Assessment (PGA) [17]. Generic and disease-specific instruments for the measurement of quality of life, such as the SF-36 and IBDQ [18], respectively, are also often included in UC clinical trials. A systematic review has demonstrated the sensitivity of the SF-36 for detecting the disease burden of UC across multiple aspects of HRQoL, in which patients with active UC were shown to experience clinically meaningful deficits in physical, emotional, and social functioning and well-being, compared with patients with inactive UC or the general population [4]. Similarly, a recent meta-analysis confirmed the sensitivity of the IBDQ for measuring treatment effects and capturing the elements of UC that have an impact on patients' daily lives [19].

It is unclear whether treatment effects on HRQoL are fully explained by changes in disease activity or if there are other mediators of treatment. Of note, evidence suggests that endoscopic targets may lack alignment with the clinical experience of the patient, as determined by instruments for the measurement of HRQoL [1, 20, 21, 22]. In particular, it has been reported that over 50% of patients whose endoscopic finding indicated moderate to severe UC reported self-perception of their disease as being “normal or in remission” [1]. Additionally, endoscopic remission has been shown to correlate well with improvements in the Mayo rectal bleeding subscore but not the stool frequency subscore [21, 22].

This post hoc analysis of data from OCTAVE Induction 1 and 2 explores the interrelationships among tofacitinib treatment, SF-36/IBDQ domains, and disease activity (as measured by Mayo subscores) using a mediation modeling framework [23, 24, 25]. The objective of this analysis was to specifically determine (1) whether tofacitinib treatment directly contributes to all changes in SF-36 and IBDQ domain scores in patients with UC outside of any changes in Mayo subscores (direct path; representing all unobserved mediators), (2) whether changes in SF-36 and IBDQ domain scores are merely a consequence of Mayo subscore changes (indirect path; fully mediated by Mayo subscores), or (3) whether changes in SF-36 and IBDQ domain scores are the result of a combination thereof.

## Materials and Methods

### Patients and Study Design

Data were pooled from OCTAVE Induction 1 and 2, two randomized, double-blind, placebo-controlled, identically designed induction studies of tofacitinib in adult patients with moderate to severe UC [14]. Data for OCTAVE Induction 1 and 2 were collected for patients from the following regions: Africa, Asia, Australasia, Europe, North America, and South America [14]. Patients had previously experienced treatment failure on, or intolerance to, corticosteroids, azathioprine, mercaptopurine, adalimumab, or infliximab. Additional details of inclusion and exclusion criteria and concomitant medications in the OCTAVE program have been reported previously [14]. Patients were included in this analysis if data for all components of the SF-36, IBDQ, and Mayo score were simultaneously available (reasons for not completing the measurements were not provided). Initially, patients were randomized 2:2:1 to receive tofacitinib 10 mg twice daily (BID) (*N* = 905), tofacitinib 15 mg BID (*N* = 22), or placebo (*N* = 234) for 8 weeks; the 15 mg BID dose group was discontinued in 2012 following feedback from regulatory authorities and a focus on further establishing dose-response relationships, and subsequent patients were randomized (4:1) to receive tofacitinib 10 mg BID or placebo [14]. As the sample size of the tofacitinib 15 mg group was too small to allow for meaningful comparison (*N* = 22), no separate analyses were performed to compare the tofacitinib dose groups.

The total Mayo score was determined at baseline and following 8 weeks of treatment [14]. Total Mayo scores range from 0 to 12, and scores on each of the four subscores (stool frequency, rectal bleeding, endoscopic appearance according to centrally assessed endoscopic subscores [sigmoidoscopy], and PGA) range from 0 to 3, with higher scores indicating more severe disease [17].

The SF-36 (version 2) was self-administered by patients at baseline and at week 8 [16]. The SF-36 assesses eight domains of functional health (physical functioning, role-physical, bodily pain, general health, vitality, social functioning, role-emotional, and mental health), which are scored from 0 (worst possible) to 100 (best possible) and then standardized into norm-based T-scores with a mean of 50 and a standard deviation of 10 for the US general population [26, 27].

The IBDQ was the least time-consuming HRQoL measure used in the OCTAVE studies. IBDQ scores were measured at an additional timepoint as the questionnaire is shorter than the SF-36 and also self-administered, therefore minimizing the patient burden [28]. Patient data were collected at baseline and at weeks 4 and 8 [14, 16]. The IBDQ provides a total score (ranging from 32 to 224; higher scores indicate better HRQoL) and four domain scores: bowel symptoms (10 items, scored from 10 to 70), systemic symptoms (5 items, scored from 5 to 35), emotional function (12 items, scored from 12 to 84), and social function (5 items, scored from 5 to 35) [18, 29, 30]. The full list of variables included in the analysis is shown in Table 1. All variables were considered continuous (with independent treatment variables represented by values of either 0 or 1).

### Outcomes and Mediation Modeling

Two mediation models were specified in this analysis, following previously established methods [23, 24, 25, 31, 32]. In the first model, stool frequency, rectal bleeding, endoscopic appearance, and PGA served as mediators between the binary treatment variable (active treatment [tofacitinib 10 or 15 mg BID] vs. placebo) and the eight SF-36 domain scores (physical functioning, role-physical, bodily pain, general health, vitality, social functioning, role-emotional, and mental health) as outcomes. In the second model, stool frequency, rectal bleeding, endoscopic appearance, and PGA served as mediators between the binary treatment variable and the four IBDQ domain scores (bowel symptoms, systemic symptoms, emotional function, and social function) as outcomes. Both models were based on all available data collected at week 8. We posit that by week 8, all processes were in a pseudo-steady state and the system could be considered to have reached an approximate state of equilibrium.

The SF-36 model included all SF-36 domains simultaneously, and the IBDQ model included all IBDQ domains simultaneously. Initial models hypothesized that all four Mayo subscores serve as mediators on the same level. However, the data from these initial models indicated that endoscopic appearance, as determined by sigmoidoscopy, should appear causally prior to the other three Mayo subscores (online suppl. Fig. 1–4; for all online suppl. material, see www.karger.com/doi/10.1159/000528788).

Online supplementary Figures 5 and 6 show the results for the standardized path coefficients in the initial models. It was anticipated that the coefficients should have been negative, indicating that a higher endoscopic subscore (i.e., disease worsening, as measured using sigmoidoscopy) would lead to worsening of the SF-36 and IBDQ domain scores. However, the positive findings meant that a higher endoscopic subscore (i.e., disease worsening) led to improvements in the SF-36 and IBDQ domain scores, which is illogical. All other paths were logical in terms of the relationships between the variables in the model.

**Fig. 1:**
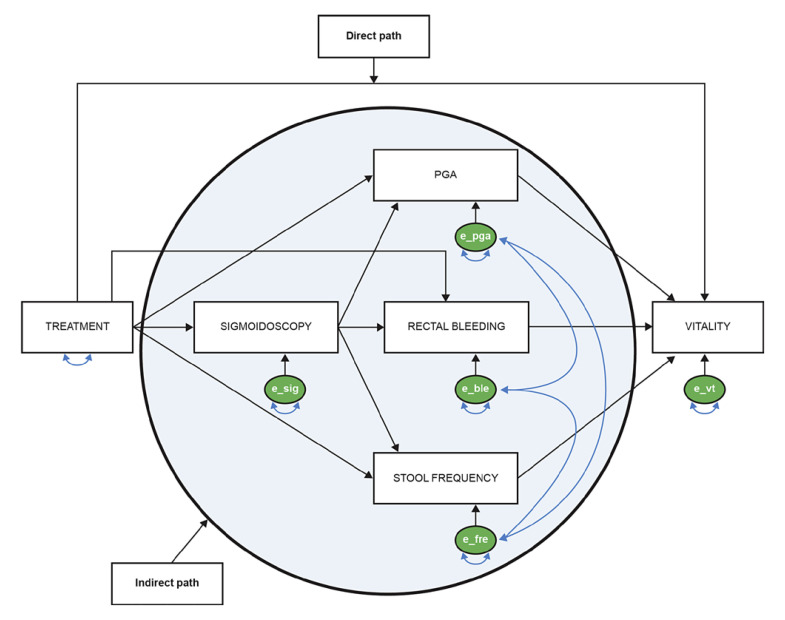
Fragment of the final respecified mediation model, using the SF-36 vitality domain as an example. This illustration is an example using the SF-36 vitality domain as the eventual outcome. e_sig, e_pga, e_ble, e_fre, and e_vt represent error terms associated with sigmoidoscopy, PGA, rectal bleeding, stool frequency, and vitality, respectively. A two-headed arrow onto itself represents variance, while a two-headed arrow between different error terms represents covariance (allowing their respective Mayo subscores to covary). PGA, Physician Global Assessment; SF-36, Short Form-36 Health Survey.

Based on this finding, the models were respecified, and a fragment of a respecified model is shown in Figure 1, using the SF-36 vitality domain as an example. The final respecified mediation models are summarized in online supplementary Figures 7 and 8. Proportions were calculated based on the path coefficients and formulas used to estimate the proportions of the treatment effects through the different paths (online suppl. Fig. 9). *p* values were calculated using the TESTFUNC function in the SAS CALIS procedure [33]. No imputation of missing data was applied.

### Direct and Indirect Path Modeling

In each model, the direct path represents the direct path from treatment to outcomes (either SF-36 or IBDQ domains, depending on which of the two models was considered). The indirect path represents the path from treatment to Mayo subscores and then to outcomes (either SF-36 or IBDQ domains).

## Results

### Baseline Demographics and Clinical Characteristics

Of the 1,161 randomized patients with moderate to severe UC, pooled from the OCTAVE Induction 1 and 2 studies (placebo, *N* = 234; tofacitinib 10 mg BID, *N* = 905; tofacitinib 15 mg BID, *N* = 22), 1,073 patients had sufficient data to be included in the SF-36 model and 1,079 patients had sufficient data to be included in the IBDQ model. Baseline demographics and characteristics were generally similar between patients in the tofacitinib (10 or 15 mg BID) and placebo groups (see online suppl. Table 1, which shows the baseline demographics and characteristics of patients in OCTAVE Induction 1 and 2).

### Mediation Effects of Mayo Subscores on SF-36 Domain Scores

**Fig. 2:**
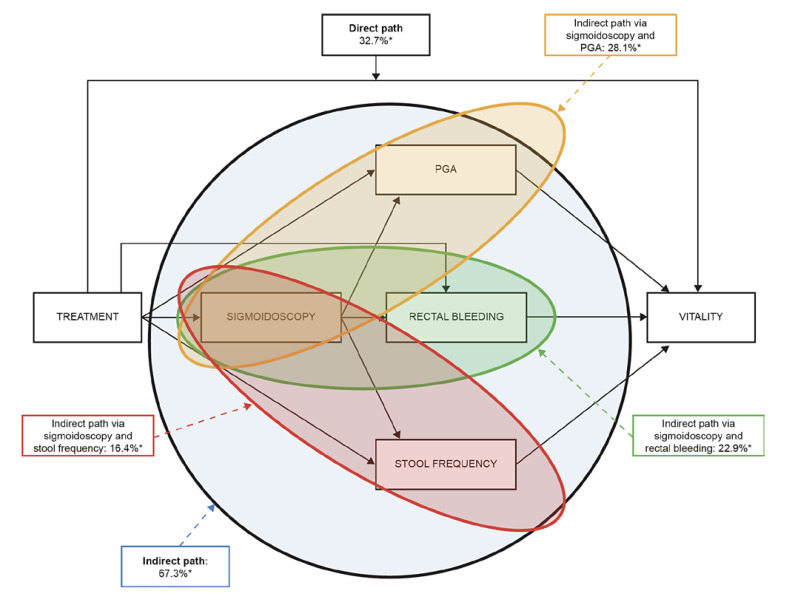
Mediation effects of treatment overall and via individual Mayo subscores, using the SF-36 vitality domain as an example. *p < 0.05. The sum of the three pairs of indirect effects (28.1% + 22.9% + 16.4%) equals the total indirect path (67.3%) before rounding; the sum of direct and indirect effects (32.7% + 67.3%) equals 100%. PGA, Physician Global Assessment; SF-36, Short Form-36 Health Survey.

The majority of the treatment effect on SF-36 domain scores was mediated by Mayo subscores. For all SF-36 domains, the overall indirect path (i.e., the pathway from treatment to Mayo subscores and then to each SF-36 domain) through Mayo subscores was significant (all *p* < 0.05) and estimated to explain between 65.6% (bodily pain) and 92.9% (mental health) of the total effect of treatment on SF-36 domain scores (Table 2). The overall indirect path was further split to assess the contributions of each Mayo subscore, as illustrated graphically in Figure 2 using the SF-36 vitality domain as an example and summarized for all SF-36 domains in Table 2. This model assumed that for indirect paths, treatment causes changes in sigmoidoscopy, which in turn causes changes in PGA/rectal bleeding/stool frequency, which in turn causes changes in SF-36 domain scores. Rectal bleeding and PGA contributions were statistically significant for all SF-36 domains (*p* < 0.05).

For the SF-36 domains of bodily pain (34.4%), role-physical (31.2%), and vitality (32.7%), the direct paths (i.e., the pathways from treatment directly to each SF-36 domain outside any effect from changes in Mayo subscores) were also significant (all *p* < 0.05). In these cases, treatment was directly associated with changes in these domain scores related to factors other than Mayo subscores. No other direct effects were statistically significant.

### Mediation Effects of Mayo Subscores on IBDQ Domain Scores

The majority of the treatment effect on IBDQ domain scores was mediated by Mayo subscores. For all IBDQ domains, the overall indirect path (i.e., the pathway from treatment to Mayo subscores and then to each IBDQ domain) through Mayo subscores was significant (all *p* < 0.05) and explained up to 84.7% (emotional function) of the total effect of treatment on IBDQ domain scores (Table 3). The indirect paths were further split to assess the contributions of each Mayo subscore, as summarized for all domains in Table 3. This model assumed that for indirect paths, treatment causes changes in sigmoidoscopy, which in turn causes changes in PGA/rectal bleeding/stool frequency, which in turn causes changes in IBDQ domain scores. For individual Mayo subscores, the indirect paths were also all significant for all IBDQ domains (all *p* < 0.05) (Table 3).

For the IBDQ domains of bowel symptoms (21.0%), social function (27.7%), and systemic symptoms (28.4%), the direct paths (owing to factors other than Mayo subscores) were also significant (all *p* < 0.05) and explained the remaining total effect of treatment. The largest direct effects were observed for the systemic symptoms and social function domains; the smallest direct effect was observed for the emotional function domain (15.3%; *p* = 0.29).

### Causal Relationship between Treatment and Mayo Subscores

**Fig. 3:**
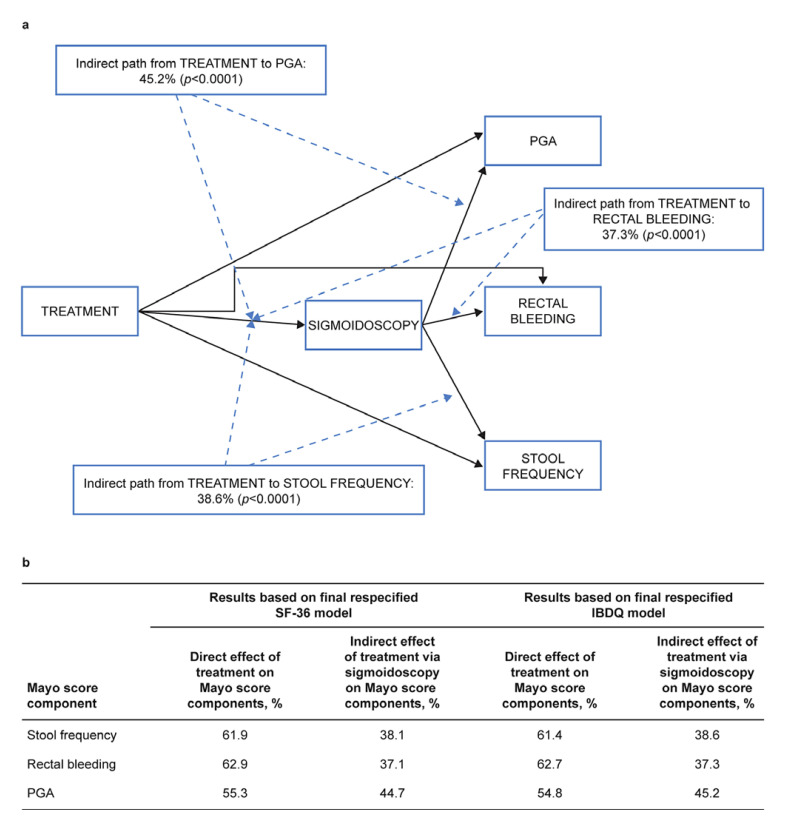
**a** Causal relationship between treatment and Mayo subscores. **b** Direct and indirect (mediated through sigmoidoscopy) effects of tofacitinib on PGA/rectal bleeding/stool frequency. All values were statistically significant (*p* < 0.0001). IBDQ, Inflammatory Bowel Disease Questionnaire; PGA, Physician Global Assessment; SF-36, Short Form-36 Health Survey.

The endoscopic appearance of the colon, as determined by sigmoidoscopy, is considered to be a causal effect for the other three Mayo score items. Therefore, the related fragment of the overall mediation model could be “extracted” for a more detailed examination as it was implied and shown as part of the respecified models. Therefore, it could be assumed that tofacitinib treatment causes changes in sigmoidoscopy, which in turn causes changes in PGA/rectal bleeding/stool frequency (i.e., indirect effects of tofacitinib treatment on PGA/rectal bleeding/stool frequency via sigmoidoscopy), and tofacitinib treatment also causes direct changes in PGA/rectal bleeding/stool frequency (shown in Fig. 3a). Based on the results of the final overall respecified model, the direct and indirect (via sigmoidoscopy) effects of tofacitinib treatment on rectal bleeding/stool frequency/PGA could also be estimated. Examination of the indirect effect of tofacitinib treatment on PGA/rectal bleeding/stool frequency, via changes in sigmoidoscopy, showed that the indirect effect constituted 37.1–45.2% of the total effect of tofacitinib treatment on rectal bleeding/stool frequency/PGA (all *p* < 0.0001) (shown in Fig. 3b). The results for this part of the model were consistent between the SF-36- and IBDQ-based models (shown in Fig. 3b).

## Discussion

This post hoc analysis of data from OCTAVE Induction 1 and 2 examined interrelationships among tofacitinib treatment, disease activity (as measured by Mayo subscores), and SF-36/IBDQ domain scores in patients with UC. The majority of tofacitinib treatment effects on HRQoL, as measured using either the generic HRQoL instrument SF-36 or the disease-specific IBDQ, were indirect effects mediated via improvements in Mayo subscores. However, indirect effects via improvements in Mayo subscores did not fully explain the effects of tofacitinib on HRQoL as statistically significant direct effects were also observed and reported. Specifically, tofacitinib directly improved certain aspects of general health status (bodily pain, role-physical, and vitality) and disease-specific HRQoL (bowel symptoms, systemic symptoms, and social function) outside the benefit of improving stool frequency, rectal bleeding, endoscopic appearance, or PGA, as measured by the Mayo score. This suggests that the treatment effect of tofacitinib on HRQoL is due to mediators beyond the Mayo subscores. For example, a cross-sectional study of patients with inflammatory bowel disease in the USA found that high levels of stress were predictive of low HRQoL; thus, high levels of stress could be considered an additional mediator of the relationship between tofacitinib treatment and patient-reported outcomes [34]. It was also determined that the Mayo endoscopic subscore, assessed via sigmoidoscopy, resulted in a causal effect for the other subscores of the Mayo score (rectal bleeding, stool frequency, and PGA).

Guidance issued by the US Food and Drug Administration in 2016, and by the European Medicines Agency in 2018, recognizes the importance of HRQoL outcomes by recommending HRQoL measurements as key to the assessment of the signs and symptoms of UC, alongside clinician-reported endoscopic and histologic evaluation [6, 35]. The use of HRQoL assessments is also encouraged across clinical trials [6]. Increasingly, practitioners are being encouraged to assess UC disease severity beyond its clinical symptoms and more in terms of patient-reported outcomes and HRQoL measures [36]. The use of generic HRQoL scales (e.g., the SF-36) to evaluate patient perspectives of disease impact may hold some advantages over disease-specific patient-reported outcome measures (e.g., the IBDQ), specifically with respect to their ability to capture a broader array of health concepts, and to offer comparisons with people outside of the disease population [4, 5]. Conversely, it should also be noted that disease-specific measures are often more responsive to improvements in clinical and endoscopic disease activity in patients with UC [4]. This increased responsiveness could reflect the integration of clinical and endoscopic disease activity measures that include UC symptoms as core domains into disease-specific HRQoL measures; thus, when symptoms are alleviated, an improvement in the HRQoL score is observed. To account for this, clinical trials should consider including HRQoL measures that are not directly impacted by symptom improvement.

Mediation modeling frameworks have been used previously to determine the associations between different exposures and treatment satisfaction among patients with inflammatory bowel disease. A post hoc analysis of data from a double-blind, placebo-controlled phase 2 trial used two mediation models, a clinician-reported outcomes-based model (which used the Mayo subscores as the mediator) and a patient-reported outcomes-based model (which used the IBDQ score domains as the mediator) to investigate the direct and indirect effects of tofacitinib treatment on patient satisfaction among patients with UC. The study found that in the clinician-reported outcomes-based model, the effect of tofacitinib treatment on patient satisfaction was predominantly mediated by improvements in the Mayo subscores; however, in the model with patient-reported mediators, the effect of tofacitinib treatment on patient satisfaction was only partially mediated by improvements in the IBDQ domains [37].

The interpretation of this analysis was limited by biases that may influence patient-reported outcomes data, such as patient tendencies to select values at the extremes of HRQoL scales, regardless of the item content (extreme response style) [38]. However, these data are still valuable as they reflect the patients' perceptions of their HRQoL at the time of self-reporting. As all of the outcome measures used in this analysis (Mayo score, SF-36, and IBDQ) include some form of patient-reported data, all of the measures were subject to similar biases.

Furthermore, the results of this analysis must be interpreted with the understanding that no technique, including mediation analyses and other forms of structural equation models, can determine causation. The purpose of this mediation analysis was to quantify the hypothesized causal relationships (e.g., changes in disease activity affect SF-36/IBDQ domain scores). Mediation modeling often requires multiple iterations since assumptions must be refined if the results of a mediation model contradict the initially hypothesized model. Even if the results of a mediation model are consistent with the hypothesized relationships between model elements, this does not prove causation; instead, it shows that the assumptions made may be valid. Additionally, inclusion of data from patients from various countries and regions who were enrolled in OCTAVE Induction 1 and 2 may have been a potential confounder in this analysis, and the factors that mediate the effects of treatment on HRQoL may vary across different geographic regions.

In conclusion, the results of these analyses indicate that Mayo scores and Mayo subscores are significant but incomplete contributors to the effects of tofacitinib on HRQoL in patients with moderate to severe UC. The results also indicate that additional mechanisms and/or pathways can affect how tofacitinib influences HRQoL outcomes and that HRQoL measurements may play an important role in the assessment of the signs and symptoms of UC. These results reinforce the value of clinicians using patient-reported HRQoL measures in addition to clinical measures, such as the Mayo score and its subscores, when assessing treatment outcomes in patients with UC. Additionally, prospectively designed studies are required to more fully elucidate other mediators of tofacitinib treatment effects on HRQoL, such as the cognitive-affective processes that may influence patient-reported outcomes.

## Statement of Ethics

The OCTAVE Induction 1 and 2 protocols were approved by the institutional review board or independent Ethics Committee at each participating center. All studies were conducted in compliance with the Declaration of Helsinki and the International Council for Harmonisation Good Clinical Practice Guidelines and were approved by the Institutional Review Boards and/or independent Ethics Committees at each of the investigational centers or a central Institutional Review Board (online suppl. Table 2). All patients provided written informed consent.

## Conflict of Interest Statement

Marla C. Dubinsky: consultancy fees and research grants from Pfizer Inc. Alessandro Armuzzi: consultancy fees from AbbVie, Allergan, Amgen, Arena, Biogen, Bristol Myers Squibb, Celgene, Celltrion, Eli Lilly, Ferring Pharmaceuticals, Gilead Sciences, Janssen, MSD, Mylan, Pfizer Inc, Roche, Samsung Bioepis, Sandoz, and Takeda; lecturing fees from AbbVie, Amgen, Biogen, Bristol Myers Squibb, Chiesi, Ferring Pharmaceuticals, Gilead Sciences, Janssen, MSD, Mitsubishi Tanabe, Nikkiso, Novartis, Pfizer Inc, Roche, Samsung Bioepis, Sandoz, Takeda, and TiGenix; research grants from MSD, Pfizer Inc, and Takeda. Krisztina B. Gecse: research support from Celltrion, Galapagos, and Pfizer Inc; and speakers' honoraria and/or consulting fees from AbbVie, Arena Pharmaceuticals, Celltrion, Ferring Pharmaceuticals, Galapagos, Gilead Sciences, Immunic Therapeutics, Janssen, Novartis, Pfizer Inc, Samsung Bioepis, Takeda, and Tillotts. Thomas Ullman: no conflicts of interest to disclose. Andrew G. Bushmakin, Marco DiBonaventura, Joseph C. Cappelleri, Susan B. Connelly, John C. Woolcott, and Leonardo Salese: employees and stockholders of Pfizer Inc.

## Funding Sources

This study was sponsored by Pfizer. Medical writing support, under the direction of the authors, was provided by Eleanor Finn, PhD, on behalf of CMC Connect, a division of IPG Health Medical Communications, and Eric Comeau, CMC Connect, and was funded by Pfizer, New York, NY, USA in accordance with Good Publication Practice (GPP 2022) guidelines (Ann Intern Med 2022; 175: 1298–1304).

## Author Contributions

Marla C. Dubinsky, Alessandro Armuzzi, Andrew G. Bushmakin, Marco DiBonaventura, Joseph C. Cappelleri, and Leonardo Salese were involved in the conceptualization of the study/analyses. Andrew G. Bushmakin and Joseph C. Cappelleri performed the formal analyses. Marla C. Dubinsky, Alessandro Armuzzi, Krisztina B. Gecse, Thomas Ullman, Andrew G. Bushmakin, Marco DiBonaventura, Joseph C. Cappelleri, Susan B. Connelly, John C. Woolcott, and Leonardo Salese contributed to data interpretation and writing − review and editing. All the authors read and approved the final version of the manuscript and agree to be accountable for all aspects of the work.

## Data Availability Statement

The datasets generated and analyzed during the current study are not publicly available in order to ensure the protection of patient privacy in compliance with the EU General Data Protection Regulation (GDPR) but are available upon reasonable request to the corresponding author.

## Supplementary Material

Supplementary data

## Figures and Tables

**Table 1:** List of variables included in the mediation analyses

Exposure	Mediators	Outcomes	
		SF-36 model	IBDQ model
Tofacitinib 10 and 15 mg BID Placebo	Endoscopic subscore Rectal bleeding subscore Stool frequency subscore PGA	Physical functioning Role-physical Bodily pain General health Social functioning Role-emotional Mental health	Bowel symptoms (10 items) Systemic symptoms (5 items) Emotional function (12 items) Social function (5 items)

BID, twice daily; IBDQ, Inflammatory Bowel Disease Questionnaire; PGA, Physician Global Assessment; SF-36, Short Form-36 Health Survey

**Table 2:** Direct and indirect effects of tofacitinib versus placebo on SF-36 domains, as a percentage of the total treatment effect

SF-36 domain	Mediation effects				
	Direct effect, % (95% CI)^a^	Indirect effect via Mayo subscores^b^			
		Overall indirect effect, % (95% CI)	Sigmoidoscopy and stool frequency, % (95% CI)	Sigmoidoscopy and rectal bleeding, % (95% CI)	Sigmoidoscopy and PGA, % (95% CI)
Bodily pain	34.4 (10.8, 58.0)	65.6 (42.0, 89.2)	16.4 (5.2, 27.6)	26.3 (13.9, 38.8)	22.9 (10.2, 35.6)
General health	23.6^ns^ (−10.2, 57.3)	76.4 (42.7, 110.2)	15.4 (1.6, 29.3)	26.6 (11.3, 42.0)	34.4 (15.0, 53.8)
Mental health	7.1^ns^ (−51.5, 65.7)	92.9 (34.3, 151.5)	19.9^ns^ (−1.7, 41.4)	27.9 (5.5, 50.3)	45.1 (11.9, 78.4)
Physical functioning	9.5^ns^ (−49.2, 68.2)	90.5 (31.8, 149.2)	17.7^ns^ (−3.6, 39.1)	26.5 (4.2, 48.7)	46.3 (11.6, 81.0)
Role-emotional	32.7^ns^ (−7.0, 72.4)	67.3 (27.6, 107.0)	13.0^ns^ (−4.5, 30.4)	19.6 (2.8, 36.3)	34.8 (9.3, 60.2)
Role-physical	31.2 (8.7, 53.7)	68.8 (46.3, 91.3)	21.5 (10.2, 32.8)	20.6 (10.5, 30.6)	26.7 (14.2, 39.3)
Social functioning	25.2^ns^ (−0.7, 51.1)	74.8 (48.9, 100.7)	24.5 (11.7, 37.3)	20.0 (9.4, 30.7)	30.2 (15.9, 44.5)
Vitality	32.7 (8.6, 56.7)	67.3 (43.3, 91.4)	16.4 (5.2, 27.5)	22.9 (11.4, 34.3)	28.1 (14.2, 42.1)

All values were statistically significant (*p* < 0.05), except those marked with “ns” (not significant). The sum of the indirect effects from sigmoidoscopy with stool frequency, rectal bleeding, and PGA equals the overall indirect effect.

a Direct path represents the direct path from treatment to outcomes (either SF-36 or IBDQ domains, depending on which of the two models was considered).

b Indirect path represents the path from treatment to Mayo subscores and then to outcomes (either SF-36 or IBDQ domains). IBDQ, Inflammatory Bowel Disease Questionnaire; PGA, Physician Global Assessment; SF-36, Short Form-36 Survey

**Table 3:** Direct and indirect effects of tofacitinib versus placebo on IBDQ domains, as a percentage of the total treatment effect

IBDQ domain	Mediation effects				
	Direct effect, % (95% CI)^a^	Indirect effect via Mayo subscores^b^			
		Overall indirect effect, % (95% CI)	Sigmoidoscopy and stool frequency, % (95% CI)	Sigmoidoscopy and rectal bleeding, % (95% CI)	Sigmoidoscopy and PGA, % (95% CI)
Bowel symptoms	21.0 (4.8, 37.2)	79.0 (62.8, 95.2)	26.8 (18.2, 35.4)	28.5 (19.7, 37.2)	23.7 (15.6, 31.8)
Emotional function	15.3^ns^ (−12.7, 43.2)	84.7 (56.8, 112.7)	25.9 (13.0, 38.7)	28.3 (15.7, 40.9)	30.6 (16.5, 44.7)
Systemic symptoms	28.4 (8.0, 48.9)	71.6 (51.1, 92.0)	24.1 (13.5, 34.7)	24.5 (14.5, 34.6)	23.0 (12.5, 33.4)
Social function	27.7 (8.8, 46.5)	72.3 (53.5, 91.2)	28.4 (17.8, 38.9)	21.3 (12.7, 30.0)	22.6 (13.1, 32.2)

All values were statistically significant (*p* < 0.05), except that marked with “ns” (not significant). The sum of the indirect effects from sigmoidoscopy with stool frequency, rectal bleeding, and PGA equals the overall indirect effect.

a Direct path represents the direct path from treatment to outcomes (either SF-36 or IBDQ domains, depending on which of the two models was considered).

b Indirect path represents the path from treatment to Mayo subscores and then to outcomes (either SF-36 or IBDQ domains). IBDQ, Inflammatory Bowel Disease Questionnaire; PGA, Physician Global Assessment; SF-36, Short Form-36 Health Survey.
