# Supplementary material for: Improvements in Disease Activity Partially Mediate the Effect of Tofacitinib Treatment on Generic and Disease-Specific Health-Related Quality of Life in Patients with Ulcerative Colitis: Data from the OCTAVE Program
Source: Dig Dis. 2023 Jan 5;41(4):604–14. doi: 10.1159/000528788 (PMC10389790; doi:10.1159/000528788)

# Supplementary Material

**Supplementary Table 1** Baseline demographics and characteristics of patients enrolled in OCTAVE Induction 1 and 2.

|  | **Tofacitinib 10 mg BID****^a^ (*N* = 905)** | **Tofacitinib 15 mg BID^a^ (*N* = 22)** | **Placebo (*N* = 234)** |
| --- | --- | --- | --- |
| Male, *n* (%) | 536 (59.2) | 12 (54.5) | 132 (56.4) |
| Age (years), mean (SD) | 41.2 (13.8) | 38.4 (12.7) | 41.1 (14.4) |
| Disease duration (years), mean (SD) | 8.1 (7.0) | 7.5 (5.6) | 8.1 (7.0) |
| BMI (kg/m^2^), mean (SD) | 24.9 (5.0) | 27.6 (6.9) | 24.6 (4.7)^d^ |
| Extent of disease, *n* (%)^b^ |  |  |  |
| Proctosigmoiditis | 132 (14.6)^c^ | 2 (9.1) | 35 (15.0)^d^ |
| Left-sided colitis | 307 (34.0)^c^ | 8 (36.4) | 76 (32.6)^d^ |
| Extensive colitis/pancolitis | 463 (51.3)^c^ | 12 (54.5) | 122 (52.4)^d^ |
| Prior TNFi failure, *n* (%) | 465 (51.4) | 10 (45.5) | 124 (53.0) |
| Corticosteroid use at baseline, *n* (%) | 412 (45.5) | 12 (54.5) | 113 (48.3) |
| Total Mayo score, mean (SD) | 9.0 (1.4)^c^ | 9.0 (1.5) | 9.0 (1.5)^d^ |

BID, twice daily; BMI, body mass index; *N*, total number of patients; *n*, number of patients in the specified category; SD, standard deviation; TNFi, tumor necrosis factor inhibitor
^a^Tofacitinib 10 and 15 mg BID dose groups were pooled in the mediation modeling analysis
^b^One patient with proctitis was enrolled into OCTAVE Induction 2 as a protocol deviation
^c^*N* = 903
^d^*N* = 233

**Supplementary Table 2. Independent Ethics Committee or Institutional Review Board details for OCTAVE Induction 1 and 2**

| **Study Details** | **Country** | **Institutional Ethics Committee or Institutional Review Board** |
| --- | --- | --- |
| OCTAVE Induction 1 (A3921094; NCT01465763) | Australia | Eastern Health Research and Ethics Committee Eastern Health Office of Research and Ethics Level 2 5 Arnold Street Box Hill, Victoria 3128 |
|  |  | Ethics Review Committee of the Sydney Local Health District (SLHD)(RPAH ZONE) Research Development Office Royal Prince Alfred Hospital Missenden Road NSW, 2050 |
|  | Austria | Ethik Kommission der Medizinischen Universitaet Wien Borschkegasse 8b/E06 Wien, 1090 |
| OCTAVE Induction 1 (A3921094; NCT01465763) [continued] | Belgium | Commissie Medische Ethiek van de Universitaire Ziekenhuizen K.U. Leuven Campus Gasthuisberg E330 Herestraat 49 Leuven, 3000 |
|  | Canada | Ethic Committee of Maisonneuve-Rosemont Hospital Pavillon Rachel-Tourigny - Porte 4158 5415 Boulevard L'Assomption Montreal, QC H1T 2M4 |
|  |  | Quorum Review Institutional Review Board Incorporated Suite 1000 1601 Fifth Avenue Seattle, WA 9810, USA |
|  |  | University of Western Ontario Office of Research Ethics Support Services Building Rm. 5150 London, ON N6G 1G9 |
| OCTAVE Induction 1 (A3921094; NCT01465763) [continued] | Canada  [continued] | Hamilton Integrated Research Ethics Board Suite 102 293 Wellington Street North Hamilton, ON L8L 8E7 |
|  |  | Conjoint Health Research Ethics Board 3rd Floor, Mackimmie Library Tower (MLT 300) 2500 University Dr. NW Calgary, AB T2N 1N4 |
|  | Colombia | Comite de Etica en Investigación en el Area de la Universidad del Norte Km. 5 Via a Puerto Colombia Barranquilla, Atlántico |
|  | Croatia | Agency for Medicinal Products and Medical Devices of Croatia, Central Ethics Committee, RoC Ksaverska cesta 4 Zagreb, 10000 |
|  | Czech Republic | Eticka komise IKEM a TN Videnska 800 Praha 4 Krc, 140 59 |
| OCTAVE Induction 1 (A3921094; NCT01465763) [continued] | Czech Republic [continued] | Eticka komise klinickeho centra ISCARE Jankovcova 1569/2C Praha 7, 170 04 |
|  |  | Eticka komise Fakultni nemocnice Hradec Kralove Sokolska 581 Hradec Kralove, 500 05 |
|  | Denmark | Den Videnskabsetiske Komité for Region Syddanmark Regionshuset, Damhaven 12 Vejle, 7100 |
|  | Estonia | Tallinn Medical Research Ethics Committee Institute of Health Department Room 24 Hiiu 42 Tallinn, 11619 |
| OCTAVE Induction 1 (A3921094; NCT01465763) [continued] | France | Comité de Protection des Personnes Ile-de-France VIII Hôpital Ambroise Paré 9 avenue Charles de Gaulle Boulogne Billancourt, 92100 |
|  | Germany | Ethik-Kommission Kiel der Medizinischen Fakultaet der Christian-Albrechts-Universitaet zu Kiel Universitaets-Kinderklinik,Schwanenweg 20 Kiel, 24105 |
|  | Hungary | Egeszsegugyi Tudomanyos Tanacs KFEB Arany Janos u. 6-8. Budapest, 1051 |
|  | Israel | Helsinki Committee Rabin Medical Center Beilinson Campus 39 Jabotinsky Street Petah Tikva, 49100 |
| OCTAVE Induction 1 (A3921094; NCT01465763) [continued] | Israel [continued] | Helsinki Committee Tel Aviv Sourasky Medical Center 6 Weizmann St. Tel Aviv, 64239 |
|  |  | Helsinki Committee-Bnai Zion Medical Center 47 Golomb St. Haifa, 31048 |
|  | Italy | Comitato Etico Indipendente del Centro di Riferimento Oncologico di Aviano Via Franco Gallini, 2 Aviano, Pordenone 33081 |
|  |  | Comitato Etico Istituto Clinico Humanitas IRCCS Via Manzoni, 56 Rozzano, Milano 20089 |
|  |  | Comitato Etico Palermo 2 Via Trabucco 180 Palermo, PA 90146 |
| OCTAVE Induction 1 (A3921094; NCT01465763) [continued] | Italy [continued] | Comitato Etico Azienda Ospedaliera Universitaria" Mater Domini Viale Europa Localita Germaneto Catanzaro, 88100 |
|  | Japan | National Hospital Organization Central Review Board 2-5-21 Higashigaoka Meguro-ku, Tokyo 152-8621 |
|  |  | Osaka City University Hospital IRB 1-5-7, Asahi-machi, Abeno-ku Osaka, Osaka 545-8586 |
|  |  | Osaka Medical College Hospital IRB 2-7, Daigaku-machi Takatsuki-shi, Osaka 569-8686 |
|  |  | Japan Community Health care Organization Tokyo Yamate Medical Center Institutional Review Board 3-22-1 Hyakunin-cho Shinjuku-ku, Tokyo 169-0073 |
| OCTAVE Induction 1 (A3921094; NCT01465763) [continued] | Japan [continued] | Tokai University Hospital Institutional Review Board 1838 Ishikawa-machi Hachioji, Tokyo 192-0032 |
|  |  | IRB of Keio University Hospital 35 Shinano-machi Shinjuku-ku, Tokyo 160-8582 |
|  |  | Kitasato University Kitasato Institute Hospital IRB 5-9-1 Shirokane Minato-ku Tokyo, 108-8642 |
|  |  | Chiba University Hospital IRB 1-8-1 Inohana Chuo-ku Chiba-shi Chiba, 260-8677 |
| OCTAVE Induction 1 (A3921094; NCT01465763) [continued] | Japan [continued] | Aichi Medical University Hospital IRB 1-1 Yazakokarimata Nagakute, Aichi 480-1195 |
|  |  | Kurume University Hospital IRB 67 Asahi-machi Kurume, Fukuoka 830-0011 |
|  |  | Keiaikai Institutional Review Board 3554-2 Babasaki Ohtsukacho Miyazaki-shi, Miyazaki 880-0951 |
|  |  | Kyoto University Hospital Institutional Review Board 54 Shogoinkawahara-cho Sakyo-ku Kyoto, Kyoto 606-8507 |
| OCTAVE Induction 1 (A3921094; NCT01465763) [continued] | Japan [continued] | Jikei University Hospital Institutional Review Board 3-19-18 Nishishinbashi, Minato-ku Tokyo, 105-8471 |
|  |  | Showa University Hospital IRB 1-5-8 Hatanodai Shinagawa-ku, Tokyo 142-8666 |
|  |  | Fukuoka University Chikushi Hospital IRB 1-1-1 Zokumyoin Chikushino, Fukuoka 8188502 |
|  |  | The Hospital of Hyogo College of Medicine IRB 1-1 Mukogawa-cho Nishinomiya, Hyogo 663-8501 |
| OCTAVE Induction 1 (A3921094; NCT01465763) [continued] | Japan [continued] | Toho University Sakura Medical Center IRB 564-1 Shimoshizu Sakura Chiba, 285-8741 |
|  |  | Tokushukai Group Institutional Review Board 1-8-7 Kojimachi Chiyoda-ku, Tokyo 102-0083 |
|  |  | Hiroshima University Hospital Institutional Review Board 1-2-3 Kasumi Minami-ku Hiroshima, Hiroshima 734-8551 |
|  |  | Yokohama Minoru Medical Clinic Institutional Review Board 1-13-8 Bessho Minami-ku Yokohama-shi, Kanagawa 232-0064 |
| OCTAVE Induction 1 (A3921094; NCT01465763) [continued] | Japan [continued] | NTT Medical Center Tokyo IRB 5-9-22 Higashi-gotanda Shinagawa-Ku, Tokyo 1418625 |
|  |  | Hokkaido P.W.F.A.C Sapporo-Kosei general Hospital Institutional Review Board 5 Higashi 8-Chome Kita3-Jo Chuo-ku Sapporo, Hokkaido 060-0033 |
|  |  | Tokyo Medical And Dental University Hospital, Faculty of Medicine Institutional Review Board 1-5-45 Yushima Bunkyo-ku Tokyo, 113-8519 |
|  |  | Shiga University of Medical Science Hospital IRB Seta Tsukinowa-cho Otsu-shi, Shiga 520-2192 |
| OCTAVE Induction 1 (A3921094; NCT01465763) [continued] | Latvia | EC for Clinical Research at Development Society Pauls Stradins Clinical University Hospital Pilsonu Street 13 Riga, LV-1002 |
|  | Netherlands | Medisch Ethische Toetsingscommissie (METC) Postbus 22.660 Amsterdam, Noord-Holland 1100 DD |
|  | New Zealand | Northern B Health and Disability Ethics Committee Ministry of Health, Ethics Department Freyberg Building 20 Aitken Street Wellington, 6011 |
|  |  | Southern Health Disability Ethics Committee Ministry of Health, Ethics Department Freyberg Building, 20 Aitken Street Wellington, 6011 |
| OCTAVE Induction 1 (A3921094; NCT01465763) [continued] | Poland | Komisja Etyk i Nadzoru nad Badaniami na Ludziach i Zwierzetach Centralnego Szpitala Klinicznego MSW w Warszawie Woloska 137 Warszawa, 02-507 |
|  | Romania | Ministerul Sanatatii, Comisia Nationala de Etica pentru Studiul pentru Studiul Clinic al Medicamentului str. Av Sanatescu nr. 48 Sector 1 Bucuresti, 011478 |
|  |  | Comisia Nationala de Bioetica a Medicamentului si Dispozitivelor Medicale Sos. Stefan cel Mare nr. 19-21 Bucuresti, Sector 2 020125 |
|  | Russian Federation | Ethics Committee at State budget Institution of Healthcare Nizhniy Novgorod Regional Clinical Hospital named after N. A. Semashko 190, Rodionova str. Nizhniy Novgorod, 603126 |
| OCTAVE Induction 1 (A3921094; NCT01465763) [continued] | Russian Federation [continued] | Ethics Committee of FSB MEI of HPE "Military Medical Academy named after S.M. Kirova" of Ministry of Defence of RF 6G, Akademika Lebedeva str. St-Petersburg, 194044 |
|  |  | Ethics Council of Ministry of Healthcare and Social Development of the Russian Federation 3, Rakhmanovskiy pereulok, GCP-4 Moscow, 127994 |
|  |  | Ethics Committee attached to Municipal Budget Institute of Healthcare "Central City Hospital of Pyatigorsk" 22 Pirogova str. Pyatigorsk, Stravropol region 357500 |
|  |  | Ethics Committee of State Budget Institution of Healthcare City Hospital #26 2 Kostyushko str. Saint Petersburg, 196247 |
| OCTAVE Induction 1 (A3921094; NCT01465763) [continued] | Russian Federation [continued] | Ethic Committee of State Budget Educational Institution of Higher Professional Education "Novosibirsk State Medical University" Ministry of Health and Social Development of the Russian Federation 52, Krasny prospect Novosibirsk, 630091 |
|  | Serbia | Ethics Committee at Limited Liability Company Medical Company "Hepatolog" 7A, Myagi str. Samara, 443093 |
|  |  | Ethics Committee, Clinical Hospital Centre Zvezdara 161 Dimitrija Tucovica St. Belgrade, 11000 |
|  |  | Ethics Committee of General Hospital "Djordje Joanovic" 5 Dr. Vase Savica St. Zrenjanin, 23000 |
| OCTAVE Induction 1 (A3921094; NCT01465763) [continued] | Serbia [continued] | Ethics Committee of Military Medical Academy Belgrade 17 Crnotravska St. Belgrade, 11000 |
|  |  | Ethics Committee of Clinical Centre of Kragujevac 30 Zmaj Jovina Street Kragujevac, 34000 |
|  | Slovakia | Eticka komisia, Urad Nitranskeho samospravneho kraja Stefanikova tr. 69 Nitra, 949 01 |
|  |  | Urad Bratislavskeho samospravneho kraja, Eticka komisia Sabinovska 16. P.O. Box 106 Bratislava 25, 820 05 |
|  |  | Eticka komisia UN Bratislava Nemocnica Stare Mesto Mickiewiczova 13 Bratislava, 813 69 |
| OCTAVE Induction 1 (A3921094; NCT01465763) [continued] | South Africa | Pharma Ethics (Pty) Ltd 123 Amcor Road Lyttelton Manor Centurion, Gauteng, 0157 |
|  |  | University of the Witwatersrand Human Research EC 8 Blackwood Avenue Johannesburg Parktown, Gauteng 2193 |
|  | Spain | CEIC Hospital Clinic I Provincial de Barcelona C/ Villarroel 170, Agencia de Ensayos Clinicos Escalera 6b-sotano Barcelona, Barcelona 08036 |
|  | Ukraine | Ethics Commission of Vinnytsia Regional Clinical Hospital for Invalids of the Great Patriotic War 109-A, Pyrogova str. Vinnytsia, 21005 |
| OCTAVE Induction 1 (A3921094; NCT01465763) [continued] | Ukraine [continued] | Central EC Ministry of Health of Ukraine 5, Narodnogo Opolchennya str. Kyiv, 03680 MSP |
|  |  | Commission of Ethic Questions of Kyiv Municipal Clinical Hospital #18 17, T.Shevchenko av. Kyiv, 01030 |
|  |  | LEC of the State Institution "L.T. Malaya Therapy Institute of National Academy of Medical Sciences of Ukraine" Department of Liver and Gastro-Intestinal Tract Diseases 2-a, Postysheva Av. Kharkiv, 61039 |
|  |  | The Central Ethics Commission of the Ministry of Health of Ukraine 5, Narodnogo Opolchennya Str. Kiev, 03680 MSP |
| OCTAVE Induction 1 (A3921094; NCT01465763) [continued] | UK | NRES Committee London-Riverside Bristol REC Centre Level 3, Block B Whitefriars Lewins Mead, Bristol BS1 2NT |
|  | USA | Baylor College of Medicine Baylor Institutional Review Board Suite 713.D One Baylor Plaza Houston, TX 77030 |
|  |  | Quorum Institutional Review Board Suite 800 1501 Fourth Ave. Seattle, WA 98101 |
| OCTAVE Induction 1 (A3921094; NCT01465763) [continued] | USA [continued] | University of Michigan Medical School Institutional Review Board (IRBMED) 2800 Plymouth Road Building 200, Room 2086 Ann Arbor, MI 48109-2800 |
|  |  | Quorum Review Institutional Review Board Incorporated Suite 1000 1601 Fifth Avenue Seattle, WA 98101 |
|  |  | Program for the Protection of Human Subjects Mount Sinai Medical One Gustave L. Levy Place New York, NY 10029 |
|  |  | Cedars Sinai Medical Center Office of Research Compliance Institutional Review Board Suite 742 8383 Wilshire Boulevard Beverly Hills, CA 90211 |
| OCTAVE Induction 1 (A3921094; NCT01465763) [continued] | USA [continued] | Human Research Protections Program (HRPP) East Campus Office Building (ECOB) 9444 Medical Center Drive First Floor La Jolla, CA 92093 |
|  |  | Washington University School of Medicine Suite 233 Human Research Protection Office 22 North Euclid Avenue Saint Louis, MO 63110 |
|  |  | Cleveland Clinic Institutional Review Board OS-1 9500 Euclid Avenue Cleveland, OH 44195 |
| OCTAVE Induction 1 (A3921094; NCT01465763) [continued] | USA [continued] | Saint Joseph Mercy Health System IRB and Research Compliance Reichert Health Building - Suite 6017 5333 McAuley Drive Ypsilanti, MI 48197 |
|  |  | Columbia University Medical Center IRB 154 Haven Avenue 1st Floor New York, NY 10032 |
| OCTAVE Induction 2 (A3921095; NCT01458951) | Australia | Royal Adelaide Hospital Research Ethics Committee Level 3, Hanson Institute, IMVS Building North Terrace Adelaide, South Australia 5000 |
|  |  | Sydney Local Health District Human Research Ethics Committee – CRGH 1st Floor - Building 75, Hospital Road Concord Repatriation General Hospital (CRGH) Concord, NSW 2139 |
| OCTAVE Induction 2 (A3921095; NCT01458951) [continued] | Australia [continued] | Monash Health Human Research Ethics Research Directorate - Monash Medical Centre 246 Clayton Road Clayton, VIC 3168 |
|  |  | ACT Health Human Research Ethics Committee ACT Health Research Office Building 10, Level 6 Canberra Hospital Yamba Drive Garran Canberra, ACT 2605 |
|  | Austria | Ethik Kommission der Medizinischen Universitaet Wien Borschkegasse 8b/E06 Wien, A-1090 |
| OCTAVE Induction 2 (A3921095; NCT01458951) [continued] | Belgium | Commissie Medische Ethiek van de Universitaire Ziekenhuizen K.U. Campus Gasthuisberg E330 Herestraat 49 Leuven, 3000 |
|  | Brazil | Comite de Etica em Pesquisa do Hospital de Clinicas de Porto Alegre - HCPA/ UFRGS Rua Ramiro Barcelos, 2350 - 2° Andar - sala 2227 Porto Alegre, Rio Grande do Sul 90035-903 |
|  |  | CONEP - Comissao Nacional de Etica em Pesquisa SEPN 510 Norte, Bloco A - 1° Subsolo Edificio Ex-INAN - Unidade II - Ministerio da Saude Brasilia, Distrito Federal 70750-521 |
| OCTAVE Induction 2 (A3921095; NCT01458951) [continued] | Canada | Montreal General Hospital - McGill University Health Centre Research Ethics Office 1650 Cedar Avenue Room C7-118 Montreal, QC H3G 1A4 |
|  |  | Quorum Review Inc. Suite 1000 1601 Fifth Avenue Seattle, WA 98101, USA |
|  |  | University of Saskatchewan BioMedical Research Ethics Board (Bio-REB) Research Ethics Office University of Saskatchewan NRC - Plant Biotechnology Research Institute 1607-110 Gymnasium Place Saskatoon, SK S7N 4J8 |
| OCTAVE Induction 2 (A3921095; NCT01458951) [continued] | Canada [continued] | University of Alberta, Health Research Ethics Board 8625 - 112 Street 308 Campus Tower Edmonton, AB T6G 1K8 |
|  | Colombia | Comite de Investigaciones y Etica en investigaciones del Hospital Pablo Tobon Uribe Calle 78B No. 69-240 Medellin, Antioquia 00000 |
|  | Croatia | Comite de Estudios Medicos S.A.S. - CREIMED S.A.S. Carrera 43A No. 34-155 - Almacentro, Piso 6 - Off. 609 Medellin, Antioquia 00000 |
|  |  | Agency for Medicinal Products and Medical Devices of Croatia, Central Ethics Committee, RoC Ksaverska cesta 4 Zagreb, 10 000 |
|  |  | Agency for Medicinal Products and Medical Devices Central Ethics Committee, RoC Ksaverska cesta 4 Zagreb, 10 000 |
| OCTAVE Induction 2 (A3921095; NCT01458951) [continued] | Czech Republic | Eticka komise IKEM a TN Videnska 800 Praha 4 Krc, 140 59 |
|  |  | Eticka komise Nemocnice Strakonice, a.s. Radomyslska 336 Strakonice, 386 29 |
|  | Denmark | De Videnskabsetiske Komitéer for Region Syddanmark Regionshuset, Damhaven 12 Vejle, 7100 |
|  | Estonia | Tallinn Medical Research Ethics Committee Institute of Health Department Room 24, 42 Hiiu Street Tallinn, 11619 |
|  | France | Comite de Protection des Personnes Ile de France VIII Hopital Ambroise Pare 9 avenue Charles de Gaulle Boulogne-Billancourt, 92100 |
| OCTAVE Induction 2 (A3921095; NCT01458951) [continued] | Germany | Ethik-Kommission Kiel der Medizinischen Fakultaet der Christian-Albrechts-Universitaet zu Kiel Universitaets-Kinderklinik,Schwanenweg 20 Kiel, 24105 |
|  | Hungary | Egeszsegugyi Tudomanyos Tanacs Klinikai Farmakologiai Etikai Bizottsaga Arany J. u. 6-8. Budapest, 1051 |
|  | Israel | Helsinki Committee-The Edith Wolfson Medical Center 62 Halohamim St. Holon, 58100 |
|  |  | Helsinki Committee Rambam Medical Center 8 Haaliya St. Haifa, 31096 |
| OCTAVE Induction 2 (A3921095; NCT01458951) [continued] | Israel [continued] | Helsinki Committee-Kaplan Medical Center POB 1 Rehovot, 76100 |
|  | Korea | Gachon University Gil Medical Center Institutional Review Board IRB Office, B2 Womens Hospital 1198 Guwol-dong Namdong-gu Incheon, 405760 |
|  |  | Hanyang University Guri Hospital Institutional Review Board 249-1, Gyomun-dong Guri-si, Gyeonggi-do 471-701 |
|  |  | Kyung Hee University Hospital Institutional Review 23 Kyungheedae-ro Dongdaemun-gu, Seoul 130-872 |
|  |  | Seoul National University Hospital IRB 101 Daehak-ro, Jongno-gu Seoul, 110-744 |
| OCTAVE Induction 2 (A3921095; NCT01458951) [continued] | Korea [continued] | Severance Hospital, Yonsei University Health System Clinical Laboratory Yonsei-ro 50-1, Seodaemun-gu Seoul, 120-752 |
|  |  | Laboratory of Samsung Medical Center 81 Irwon-ro, Gangnam-gu Seoul, 135-710 |
|  |  | Pusan National University Hospital IRB 179 Gudeok-Ro Seo-Gu Busan, 602-739 |
|  |  | Asan Medical Center Institutional Review Board 88 Olympic-ro, 43-gil Songpa-gu Seoul, 138-736 |
|  |  | CHA Bundang Medical Center, CHA University Institution Review Board 59 Yatap-ro, Bundang-gu Seongnam-si, Gyeonggi-do 463-712 |
| OCTAVE Induction 2 (A3921095; NCT01458951) [continued] | Latvia | EC for Clinical Research at Development Society Pauls Stradins Clinical University Hospital Pilsonu Street 13 Riga, LV 1002 |
|  | Netherlands | Medisch Ethische Toetsingscommissie (METC) Postbus 22660 Amsterdam, 1100 DD |
|  | New Zealand | Multi-Region Ethics Committee C/-Ministry of Health 1-3 The Terrace Wellington, 6011 Southern Health Disability Ethics Committee |
|  |  | Ministry of Health, Ethics Department Freyberg Building 20 Aitken Street Wellington, 6011 |
| OCTAVE Induction 2 (A3921095; NCT01458951) [continued] | New Zealand [continued] | Southern Health and Disability Ethics Committee C/-Medsafe, Level 6 Deloitte House 10 Brandon Street Wellington, 6011 |
|  | Poland | Komisja Etyki i Nadzoru nad Badaniami na Ludziach ul. Woloska 137 Warszawa, mazowiecke 02-507 |
|  |  | Komisja Etyki i Nadzoru nad Badaniami na Ludziach i Zwierzetach Centralnego Szpitala Klinicznego MSW w Warszawie ul. Woloska 137 Warszawa, 02-507 |
|  |  | Komisja Bioetyczna, Centralny Szpital Kliniczny MSWiA ul. Woloska 137 Warszawa, 02-507 |
| OCTAVE Induction 2 (A3921095; NCT01458951) [continued] | Romania | Ministerul Sanatatii, Comisia Nationala de Etica pentru Studiul Clinic al Medicamentului str. Av Sanatescu nr. 48 Sector 1 Bucuresti, 011478 |
|  | Russian Federation | Ethics Council of Ministry of Health and Social Development 3, Rakhmanovskiy pereulok, GSP-4 Moscow, 127994 |
|  |  | Independent Ethics Committee attached to State budget Healthcare Institution Moscow regional scientific research clinical institute named after M.F. Vladimirsky Shepkina str., 61/2 Moscow, 129110 |
|  |  | EC State Budget Educ. Inst. Of Higher Prof. Education "Yaroslavl State Medical Academy of MoH and Soc. Development of the RF 5, Revolutsionnaya str. Yaroslavl, 150000 |
| OCTAVE Induction 2 (A3921095; NCT01458951) [continued] | Russian Federation [continued] | Ethics Council of Ministry of Health and Social Development 3, Rakhmanovskiy pereulok GSP-4, Moscow, 127994 |
|  | Serbia | Ethics Committee, Clinical Centre of Vojvodina 1 Hajduk Veljkova St. Novi Sad, 21000 |
|  |  | Ethics Committee of Clinical Centre of Serbia 2 Pasterova St. Belgrade, 11000 |
|  |  | Ethics Committee Clinical Centre of Nis 48 Dr Zorana Djindjica Boulevard Nis, 18000 |
|  | Slovakia | Ethics Committee of General Hospital Subotica,Subo 3 Izvorska St. Subotica, 24000 |
| OCTAVE Induction 2 (A3921095; NCT01458951) [continued] | Slovakia [continued] | NsP Nove Mesto n. Vahom n.o., Eticka komisia M. R. Stefanika 1 Nove Mesto nad Vahom, 915 01 |
|  |  | Urad Nitrianskeho samospravneho kraja Eticka Komisia Stefanikova tr. 69 Nitra, 94901 |
|  |  | Eticka komisia Presovskeho samospravneho kraja Namestie mieru 2 Presov, 080 01  Etiska komisia, Urad Nitrianskeho samospravneho kraja Razusova 2A Nitra, 94901 |
|  | South Africa | Pharma Ethics (Pty) Ltd 123 Amcor Road Lyttelton Manor Pretoria, 0157 |
| OCTAVE Induction 2 (A3921095; NCT01458951) [continued] | Spain | CEIC del Hospital Universitario de Bellvitge. Edificio de Recerca C/ Feixa Llarga s/n L'Hospitalet de Llobregat, Barcelona 08907 |
|  |  | CEIC Hospital Clinic I Provincial de Barcelona C/ Villarroel 170. Agencia de Ensayos Clinicos Escalera 6b-sotano Barcelona, Barcelona 08036 |
|  | Taiwan | Institutional Review Board of Chung Shan Medical University Hospital No. 110 Sec. 1, Chien Kou North Road Taichung, 40201 |
|  |  | Chang Gung Medical Foundation Institutional Review Board No.199, Dunhua N Rd.,Songshan Dist. Taipei City, Taiwan 105 |
|  |  | Research Ethics Committee of National Taiwan University Hospital No.7 Chung-Shan South Road ZhongZheng District Taipei City, 10002 |
| OCTAVE Induction 2 (A3921095; NCT01458951) [continued] | Ukraine | LEC of Municipal Healthcare Institution 197 Moskovskiy Avenue Kharkiv City Clinical Hospital #2", Proctology Department Khakiv, 61037 |
|  |  | The Central Ethics Commission of the Ministry of Health of Ukraine 5, Narodnogo Opolchennya str. Kyiv, MSP 03680 |
|  |  | Ethics Commission of LLC "Medical and Diagnostic Center "Slaomed" 96-A, Khmelnytske Highway Str Vinnytsya, 21029 |
|  |  | Central Ethics Commission of Ministry of Health of Ukraine 5, Narodnogo Opochennya str. Kyiv, 03680 MSP |
|  |  | LEC of the Kyiv City Clinical Hospital #8 8, Kondratyuka Str Kyiv, 04201 |
| OCTAVE Induction 2 (A3921095; NCT01458951) [continued] | Ukraine [continued] | ETHICS Commission of Municipal. Institution."Odesa Commission of Municipal. Institution."Odesa. Regional. Clinical.Hospital" 26, Zabolotnogo str. Odesa, 65025 |
|  |  | Local Ethics Commission of Regional Municipal Institution 137,Golovna str.  Chernivtsi, 58001 |
|  | UK | NRES Committee London Riverside South West Research Ethics Committee Level 3, Block B,Whitefriars, Lewins Mead Bristol, BS1 2NT |
|  | USA | Quorum Review Institutional Review Board Suite 800 1501 Fourth Avenue Seattle, WA 98101 |
| OCTAVE Induction 2 (A3921095; NCT01458951) [continued] | USA [continued] | Quorum Review Institutional Review Board Inc. Suite 1000 1601 Fifth Avenue Seattle, WA 98101 |
|  |  | Quorum Review Inc. Suite 1000 1601 Fifth Avenue Seattle, WA 98101 |
|  |  | The Institutional Review Board of The Guthrie Clinic One Guthrie Square Sayre, PA 18840 |
|  |  | Western Institutional Review Board Suite 120 1019 39th Avenue, SE Puyallup, WA 98374-2115 |
| OCTAVE Induction 2 (A3921095; NCT01458951) [continued] | USA [continued] | Mayo Clinic Institutional Review Board 200 First Street Southwest Rochester, MN 55905 |
|  |  | Aurora IRB P.O. Box 342 W310 945 North 12th Street Milwaukee, WI 53201-0342 |
|  |  | UCSF Committee on Human Research Suite 315 - Box 0962 3333 California Street San Francisco, CA 94118 |
|  |  | Office of Human Research Protection Program Suite 211 11000 Kinross Avenue Los Angeles, CA 90095 |
| OCTAVE Induction 2 (A3921095; NCT01458951) [continued] | USA [continued] | Yale University Human Research Protection Program 55 College Street New Haven, CT 06510 |
|  |  | Chesapeake Institutional Review Board Suite 100 7063 Columbia Gateway Drive Columbia, MD 21046 |
|  |  | Western Institutional Review Board (WIRB) 3535 7th Avenue SW Olympia, WA 98502-5010 |
|  |  | Human Research Protection Program 504 Oxford House Nashville, TN 37232 |
|  |  | Cleveland Clinic Foundation IRB 9500 Euclid Avenue Cleveland, OH 44195 |

**Supplementary Fig. 1.** Summary of the initial mediation model relevant to the SF‑36 domains (to be read from left to right). e_sig, e_pga, e_ble, e_fre, e_vt, e_sf, e_rp, e_re, e_mh, e_pf, e_gh, and e_bp represent error terms associated with sigmoidoscopy, PGA, rectal bleeding, stool frequency, VT, SF, RP, RE, mental health, PF, GH, and BP, respectively. Error terms e_vt, e_sf, e_rp, e_re, e_mh, e_pf, e_gh, and e_bp are allowed to covary (generally represented by a two-headed arrow between different error terms; not shown for simplicity), meaning that the SF-36 domains are not considered as independent concepts. Error terms e_sig, e_pga, e_ble, and e_fre are allowed to covary (generally represented by a two-headed arrow between different error terms; not shown for simplicity in this figure; see also Supplementary Figure 2), meaning that sigmoidoscopy, PGA, rectal bleeding, and stool frequency are not considered as independent concepts. BP, bodily pain; GH, general health; MH, mental health; PGA, Physician Global Assessment; PF, physical functioning; RE, role-emotional; RP, role‑physical; SF, social functioning; SF‑36, Short Form‑36 Health Survey; Trt, pooled active treatments vs placebo; VT, vitality.


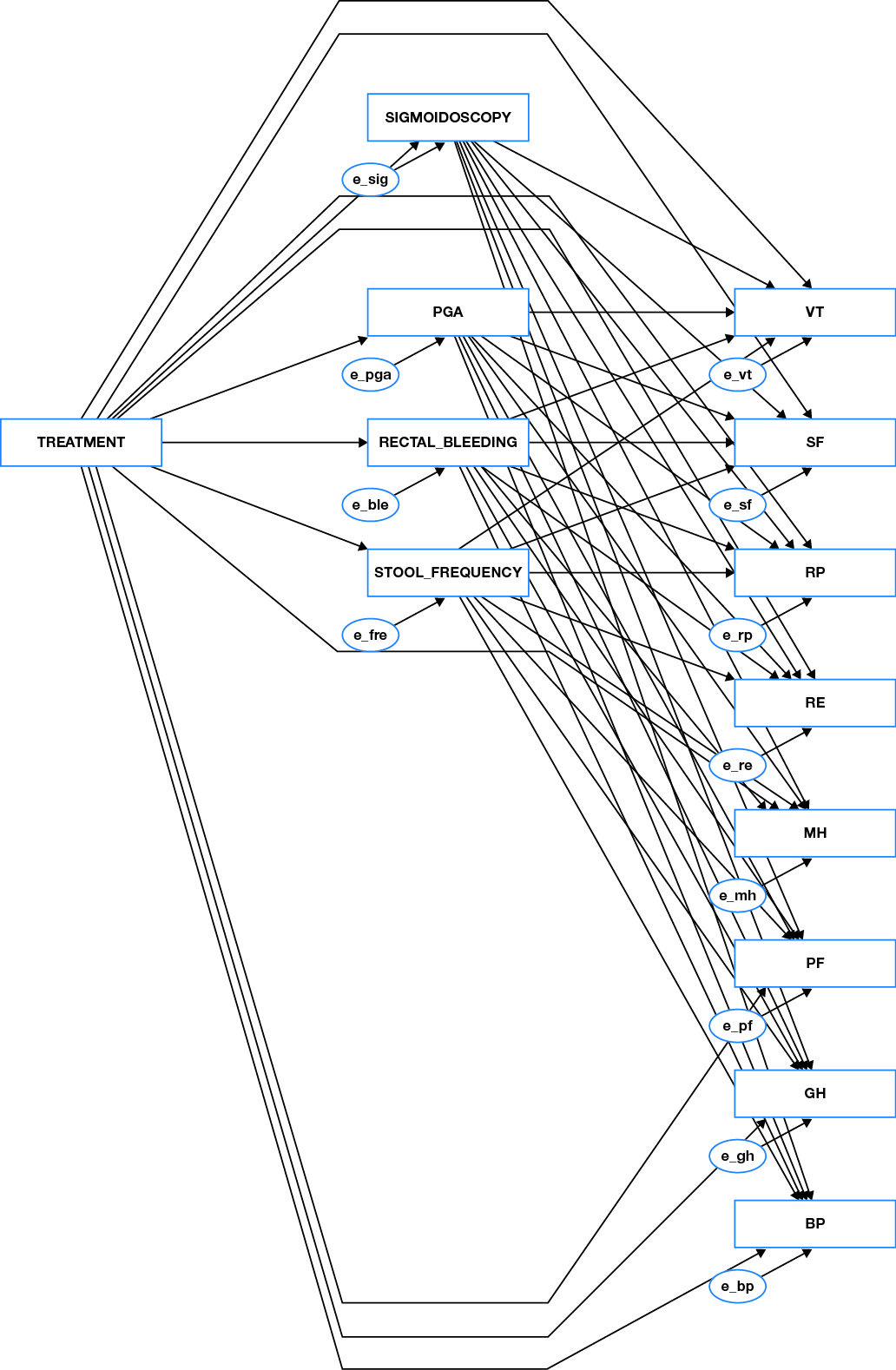


**Supplementary Fig. 2.** Fragment of the initial mediation model relevant to the SF-36 domains, using the SF-36 vitality domain as an example. This illustration is an example using the SF-36 vitality domain as the eventual outcome. e_fre, e_ble, e_pga, e_sig, and e_vt represent error terms associated with stool frequency, rectal bleeding, PGA, sigmoidoscopy, and VT, respectively. A two-headed arrow onto itself represents variance, while a two‑headed arrow between different error terms represents covariance (meaning that sigmoidoscopy, PGA, rectal bleeding, and stool frequency are not considered as independent concepts). PGA, Physician Global Assessment; SF-36, Short Form-36 Health Survey; Trt, pooled active treatments vs placebo; VT, vitality.


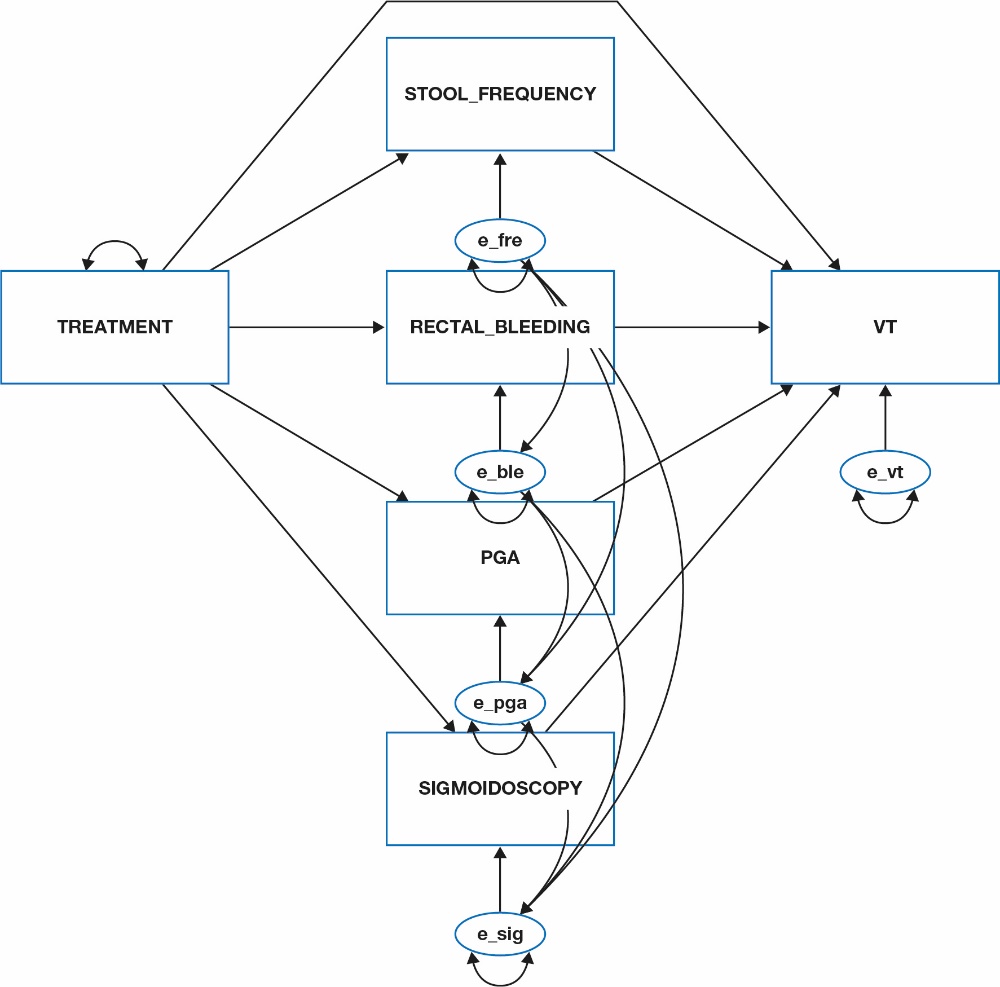


**Supplementary Fig. 3.** Summary of the initial mediation model relevant to the IBDQ domains (to be read from left to right). e_pga, e_sig, e_ble, e_fre, e_soc, e_sys, e_emo, and e_bow represent error terms associated with PGA, sigmoidoscopy, rectal bleeding, stool frequency, social function, systemic symptoms, emotional function, and bowel symptoms, respectively. Error terms e_soc, e_sys, e_emo, and e_bow are allowed to covary (generally represented by a two-headed arrow between different error terms; not shown for simplicity), meaning that the IBDQ domains are not considered as independent concepts. Error terms e_pga, e_sig, e_ble, and e_fre are allowed to covary (generally represented by a two-headed arrow between different error terms; not shown for simplicity; see also Supplementary Figure 4), meaning that PGA, sigmoidoscopy, rectal bleeding, and stool frequency are not considered as independent concepts. IBDQ, Inflammatory Bowel Disease Questionnaire; PGA, Physician Global Assessment; Trt, pooled active treatments vs placebo.


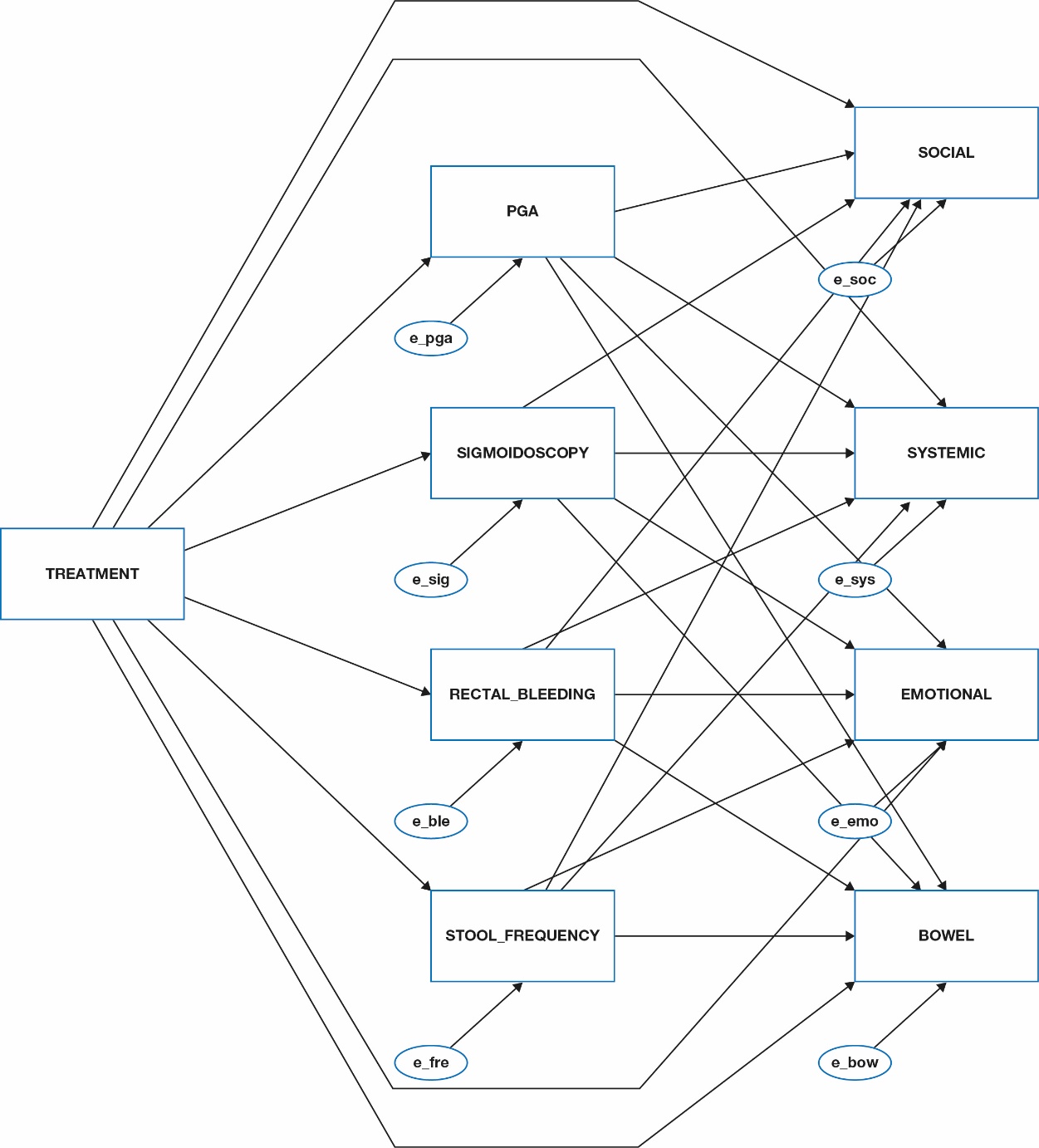


**Supplementary Fig. 4.** Fragment of the initial mediation model relevant to the IBDQ domains, using the IBDQ bowel symptoms domain as an example. This illustration is an example using the IBDQ bowel symptoms domain as the eventual outcome. e_fre, e_ble, e_pga, e_sig, and e_bow represent error terms associated with stool frequency, rectal bleeding, PGA, sigmoidoscopy, and bowel symptoms, respectively. A two-headed arrow onto itself represents variance, while a two-headed arrow between different error terms represents covariance (meaning that sigmoidoscopy, PGA, rectal bleeding, and stool frequency are not considered as independent concepts). IBDQ, Inflammatory Bowel Disease Questionnaire; PGA, Physician Global Assessment; Trt, pooled active treatments vs placebo.


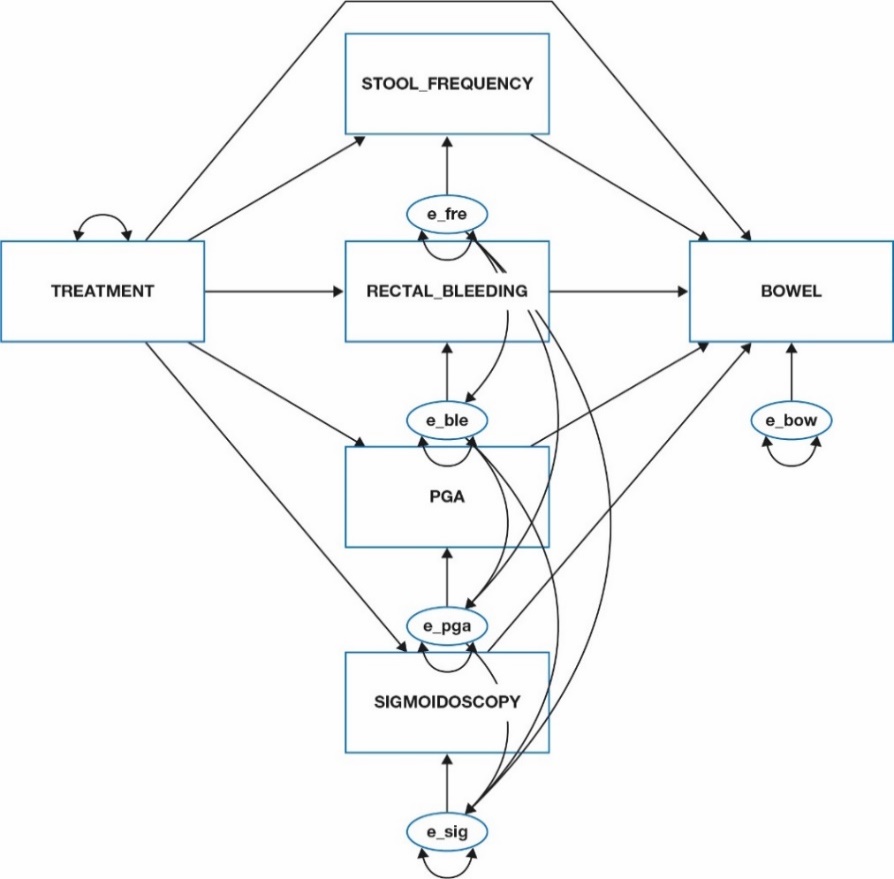


**Supplementary Fig. 5.** Results for the standardized path coefficients in the initial mediation model relevant to the SF-36 domains. It was anticipated that the coefficients (highlighted yellow) should have been negative, indicating that a higher endoscopic subscore (i.e., disease worsening; as measured using sigmoidoscopy) would lead to worsening in the SF-36 domain scores. However, the positive finding meant that a higher endoscopic subscore (i.e., disease worsening) led to improvements in the SF-36 domain scores, which is illogical. All other paths were logical in terms of the relationships between variables in the model. For example, the standardized path coefficient for the direct path from treatment to BP was positive (highlighted green), indicating that treatment directly improved BP. The direct paths from treatment to the Mayo subscores were negative (highlighted cyan), indicating that treatment directly improved the Mayo subscores. Estimates that are significant at the 0.05 alpha-level are flagged with an asterisk (*), and estimates that are significant at the 0.01 alpha-level are flagged with a double asterisk (**). BP, bodily pain; GH, general health; MH, mental health; ns, not significant (*p*>0.05); PGA, Physician Global Assessment; PF, physical functioning; RE, role-emotional; RP, role-physical; SF, social functioning; SF-36, Short Form‑36 Health Survey; Trt, pooled active treatments vs placebo; VT, vitality.


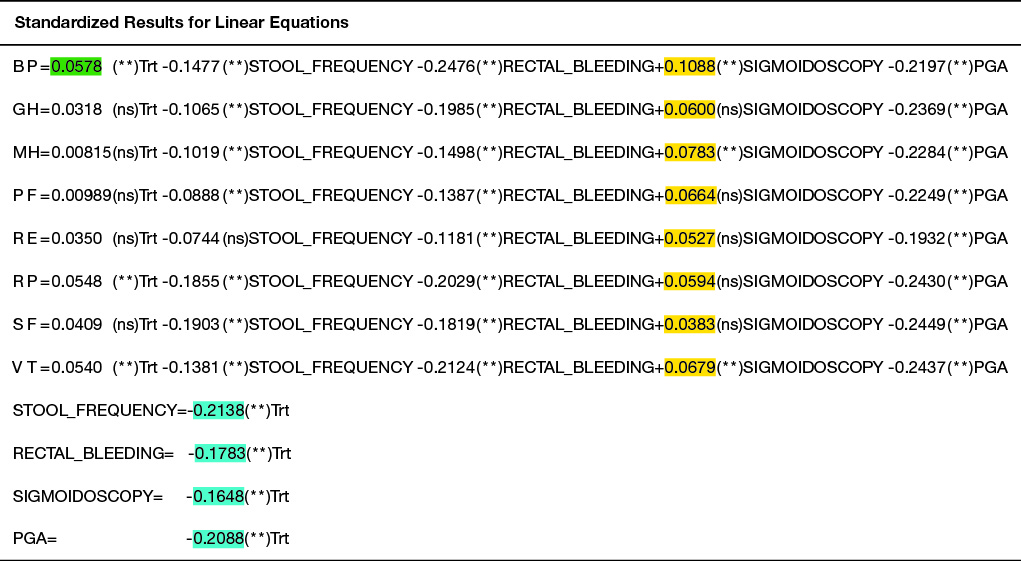


**Supplementary Fig. 6.** Results for the standardized path coefficients in the initial mediation model relevant to the IBDQ domains. It was anticipated that the coefficients (highlighted yellow) should have been negative, indicating that a higher endoscopic subscore (i.e., disease worsening; as measured using sigmoidoscopy) would lead to worsening in the IBDQ domain scores. However, the positive finding meant that a higher endoscopic subscore (i.e., disease worsening) led to improvements in the IBDQ domain scores, which is illogical. All other paths were logical in terms of the relationships between variables in the model. For example, the standardized path coefficient for the direct path from treatment to bowel symptoms was positive (highlighted green), indicating that treatment directly improved bowel symptoms. The direct paths from treatment to the Mayo subscores were negative (highlighted cyan), indicating that treatment directly improved the Mayo subscores. Estimates that are significant at the 0.05 alpha-level are flagged with an asterisk (*), and estimates that are significant at the 0.01 alpha-level are flagged with a double asterisk (**). IBDQ, inflammatory Bowel Disease Questionnaire; ns, not significant (*p*>0.05); PGA, Physician Global Assessment; Trt, pooled active treatments vs placebo.


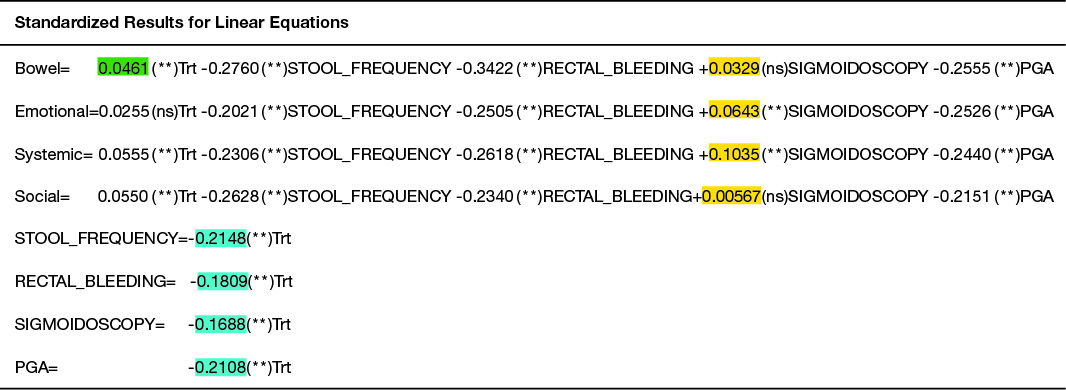


**Supplementary Fig. 7.** Summary of the final mediation model relevant to the SF‑36 domains (to be read from left to right). e_sig, e_ble, e_fre, e_pga, e_vt, e_re, e_sf, e_pf, e_rp, e_gh, e_mh, and e_bp represent error terms associated with sigmoidoscopy, rectal bleeding, stool frequency, PGA, vitality, role-emotional, social functioning, physical functioning, role‑physical, general health, mental health, and bodily pain, respectively. Error terms e_ble, e_fre, and e_pga are allowed to covary (generally represented by a two‑headed arrow between different error terms; not shown for simplicity), meaning that rectal bleeding, stool frequency, and PGA are not considered as independent concepts. Error terms e_vt, e_re, e_sf, e_pf, e_rp, e_gh, e_mh, and e_bp are allowed to covary (generally represented by a two-headed arrow between different error terms; not shown for simplicity), meaning that the SF-36 domains are not considered as independent concepts. BP, bodily pain; GH, general health; MH, mental health; PGA, Physician Global Assessment; PF, physical functioning; RE, role-emotional; RP, role‑physical; SF, social functioning; SF‑36, Short Form‑36 Health Survey; VT, vitality.


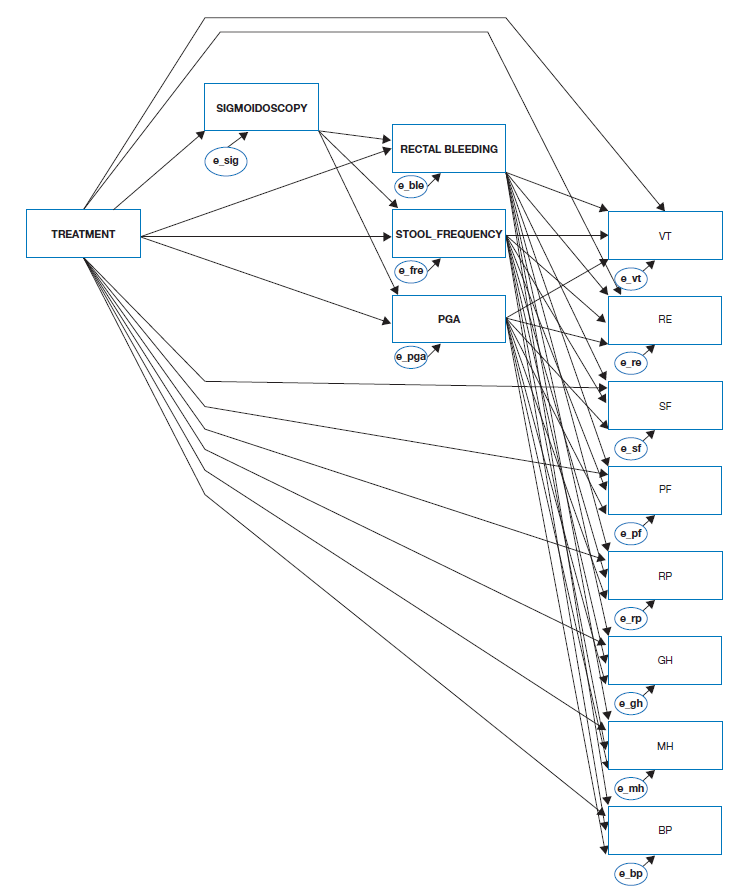


**Supplementary Fig. 8.** Summary of the final mediation model relevant to the IBDQ domains (to be read from left to right). e_sig, e_ble, e_pga, e_fre, e_sys, e_emo, e_soc, and e_bow represent error terms associated with sigmoidoscopy, rectal bleeding, PGA, stool frequency, systemic symptoms, emotional health, social function, and bowel symptoms, respectively. Error terms e_pga, e_ble, and e_fre are allowed to covary (generally represented by a two‑headed arrow between different error terms; not shown for simplicity), meaning that PGA, rectal bleeding, and stool frequency are not considered as independent concepts. Error terms e_sys, e_emo, e_soc, and e_bow are allowed to covary (generally represented by a two-headed arrow between different error terms; not shown for simplicity), meaning that the IBDQ domains are not considered as independent concepts. IBDQ, Inflammatory Bowel Disease Questionnaire; PGA, Physician Global Assessment.


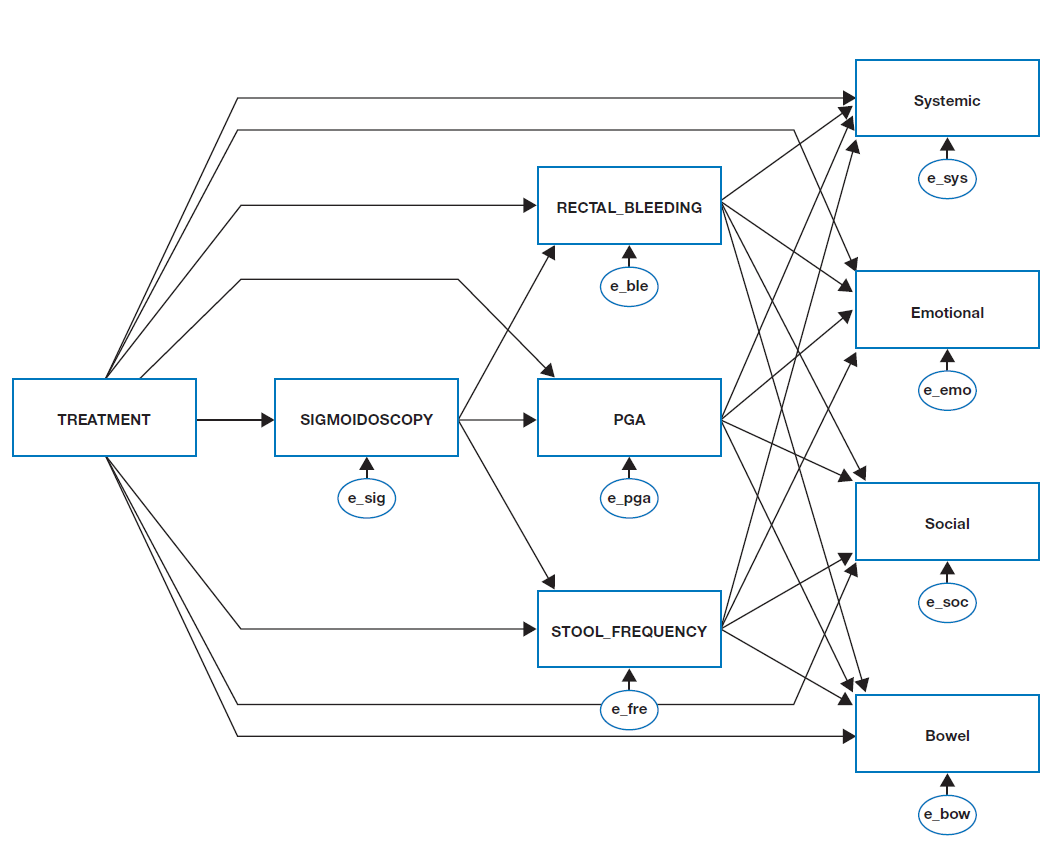


**Supplementary Fig. 9.** Fragment of the final mediation model relevant to the SF-36 domains with path coefficients, using the SF-36 vitality domain as an example. **a** This illustration is an example using the SF-36 vitality domain as the eventual outcome. Abbreviations representing the path coefficients (e.g., l_trt_vt) are shown on each line. **b** The formulas used to estimate the proportions of the treatment effect through different paths, based on the path coefficients. In the formulas, an asterisk (*) represents multiplication. PGA, Physician Global Assessment; SF-36, Short Form-36 Health Survey; Trt, pooled active treatments vs placebo; VT, vitality.


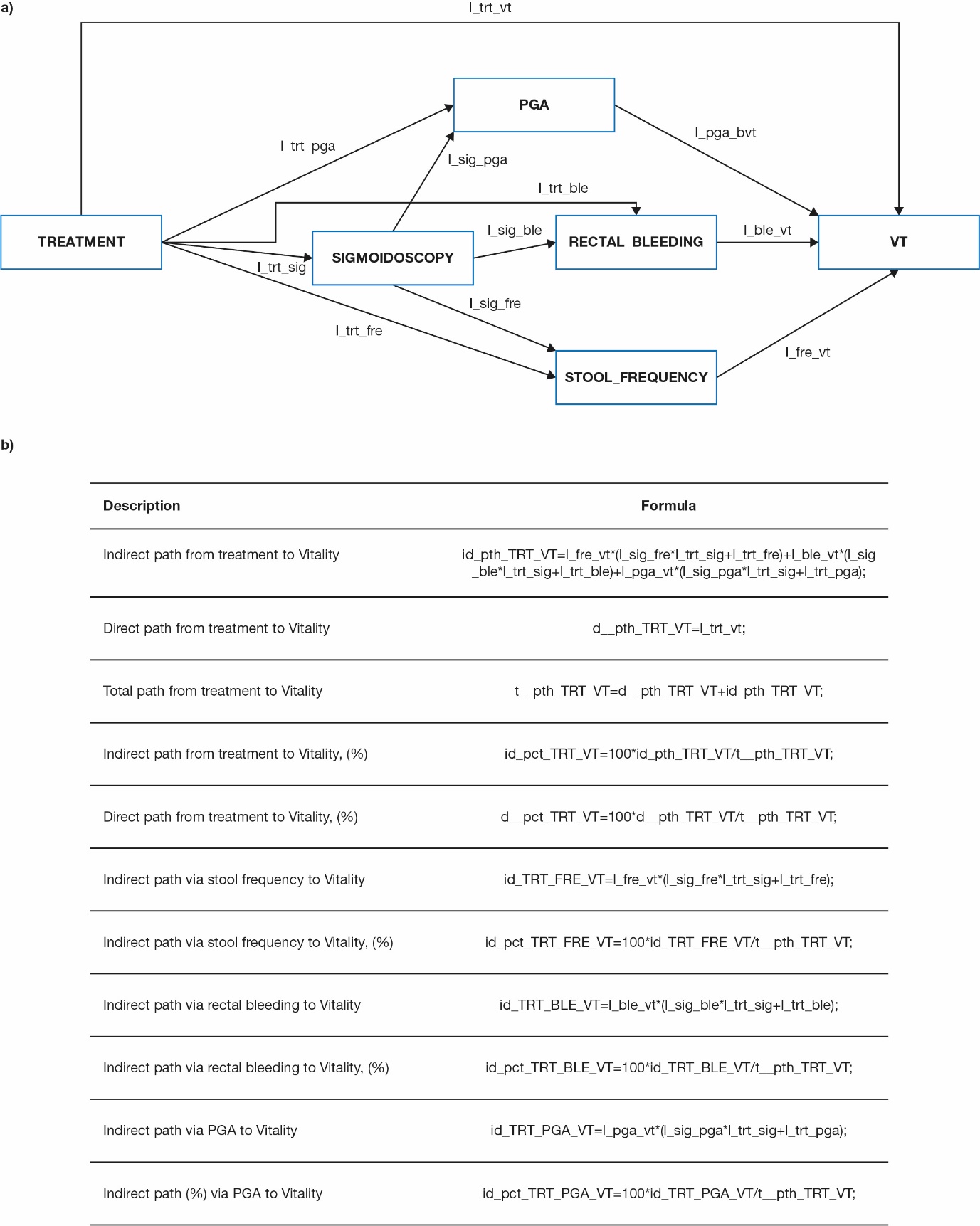

Supplement: Supplementary file 1 — Supplementary data [file ddi-0041-0604-s01.docx]
